# Engaging youth in global health and social justice: a decade of experience teaching a high school summer course

**DOI:** 10.1080/16549716.2021.1987045

**Published:** 2022-02-02

**Authors:** Heather Wipfli, Mellissa Withers

**Affiliations:** a Departments of Preventive Medicine and International Relations, University of Southern California, Los Angeles, CA, USA; b Department of Preventive Medicine, Keck School of Medicine, University of Southern California, Los Angeles, CA, USA

**Keywords:** Global health, education, social justice, high school

## Abstract

**Background:**

Education and training in global health and social justice is crucial to ensuring the next generation of health professionals are poised to tackle the pressing issues of our time.

**Objectives:**

To develop and implement an intensive summer course on global health and social justice for high school students at the University of Southern California.

**Methods:**

This paper reviews the course aims and curriculum, its implementation both onsite and online, and reports on student course evaluations and long-term student outcomes.

**Results:**

Student satisfaction with the program was extremely high, which endured when the course went virtual during the COVID-19 pandemic. The most popular elements of the course included interactive, game-based activities and guest speakers. Many students reported that the course motivated them to pursue higher education and careers in global health or related fields.

**Conclusions:**

More universities should consider offering summer programs or other extension programs targeting high school students in order to meet the increasing demand for global health education. Recommendations for implementing global health courses for younger learners include having an interdisciplinary focus with a range of topics and diverse perspectives; provision of scholarships to allow low-income students and students from abroad to participate; prioritizing the integration of active learning and experiential educational opportunities; and incorporating debriefing and reflection as integral parts of learning.

## Background

Global health is the term applied to collaborative trans-national research, training, and action for promoting health for all worldwide [1]. Global health education and training, which goes beyond core public health competencies such as biostatistics or epidemiology to include social justice and an emphasis on health as a public good [2], is crucial to ensuring the next generation of global health professionals are poised to tackle the pressing issues of our time. Universities have an important role in offering such educational programs and throughout the early 2000s global health programs expanded rapidly throughout universities in the US and abroad, driven in large part by student demand and interest in the field [3–6]. Today, global health is an undergraduate major/minor/certificate at more than four hundred universities throughout the US, enrolling thousands of students annually [7]. These programs are often attractive to college-bound students interested in studying the intersection of health care, politics, sustainability, and social justice. Notably, this interest is likely to increase in the wake of the COVID-19 pandemic.

Since 2011, the University of Southern California (USC) has offered an intensive summer course for high school students in global health and social justice. The course surveys the broad foundations of global health ranging from disease characteristics and epidemiology to social determinants of health, global governance and ethics. Over time, the course has added to its content focused on social justice and racial inequities in health as representative of broader structural racism within the United States (US) and power relationships between low- and middle-income countries (LMICs) and high-income countries (HICs). The course curriculum incorporates numerous hands-on activities, guest speakers, and experiential visits to engage the young learners in the diverse topics and expose them to future career opportunities. The core aim of the course is to leave the students motivated to learn more and inspired to consider careers that contribute to improved health equity in the US and around the world. This paper outlines how we, the authors, developed the course aims and curriculum, implemented it both onsite and online, and reports on student evaluations and long-term student outcomes. The paper concludes with lessons we have learned and our recommendations for introducing young learners to some of the most pressing and challenging issues of our time. As evidenced through the USC course experience, introducing young learners to the broad range of global health approaches and career opportunities can play a significant role in motivating them to seek more related education and pursue a relevant profession.

## Background: course design and implementation

The course was designed for, and offered through, the ‘Summer @ USC’ program [8]. The four-week, for-credit, college-immersion program offers high school students university experience in a subject area of their choice. The program offers over two dozen different courses allowing students to explore new areas of study or build on their high school coursework. Such summer programs located on college campuses targeting high school students have grown quickly over the past few decades to the point where there are now hundreds of programs and thousands of courses to choose from [9]. These programs promise to provide students with an approximate college experience by offering classes taught by professors and housing students in dorms (although commuting options also exist). And while many programs, including that of USC, offer college credit for completion, there is also a recognition that the students are on ‘break’ and need a fair amount of entertainment and socialization built into the program, both in and out of class. In class, this often translates into interactive exercises, game-based learning, and immersive field trips. Out of class this frequently takes the form of networking events, trips to amusement parks, and dorm parties. Students in the Summer @ USC program come from throughout the US and internationally and represent various high school grades, ages (14–17). Reflective of the high tuition, they are generally from high-income households and tend to be well-travelled. Approximately 150 students have participated in the global health course over the past decade. The course faculty at USC receive additional compensation for teaching the course and are provided a small budget for course materials and activities.

### Course learning objectives

In designing a summer course in global health, we set out a number of learning objectives for our students (Box 1). A central goal of the course is to expose students to the multi-disciplinary nature of global health, including curriculum from biology, epidemiology, psychology, engineering, philosophy, law, international relations, theater and cinematic arts. These disciplines are not only reflective of those engaged in the global health field but make the course interesting and applicable to a wide range of young learners who are not yet certain about the direction the want to take their future career or education. In other words, regardless of a student’s primary interests, no one is bored for long.

Over time, the course expanded (for the first two years the course was only two weeks long) and shifted to include a greater focus on social justice and racial inequities in health as representative of broader structural inequities and racism within the US and between low- and middle-income countries (LMICS) and high-income countries. This focus was in part motivated by increased public discourse and awareness of issues related to power, privilege and race among high schoolers and a desire to further stress global citizenship. The course now incorporates data sets stratified by race, discussions of economic and racial disparities in access to care, and readings on police and other forms of political violence.

In 2020, the course was pivoted slightly to focus heavily on the concurrent COVID-19 outbreak. The pandemic primarily served as a core case study to illustrate the same challenges and trends covered in the class before the crisis, including tracking and tracing disease outbreaks, evaluating global governance mechanisms to respond to pandemics, assessing inequities in access to health care goods and services, and debating ethical issues related to research and serving vulnerable populations.

**Box #1.**
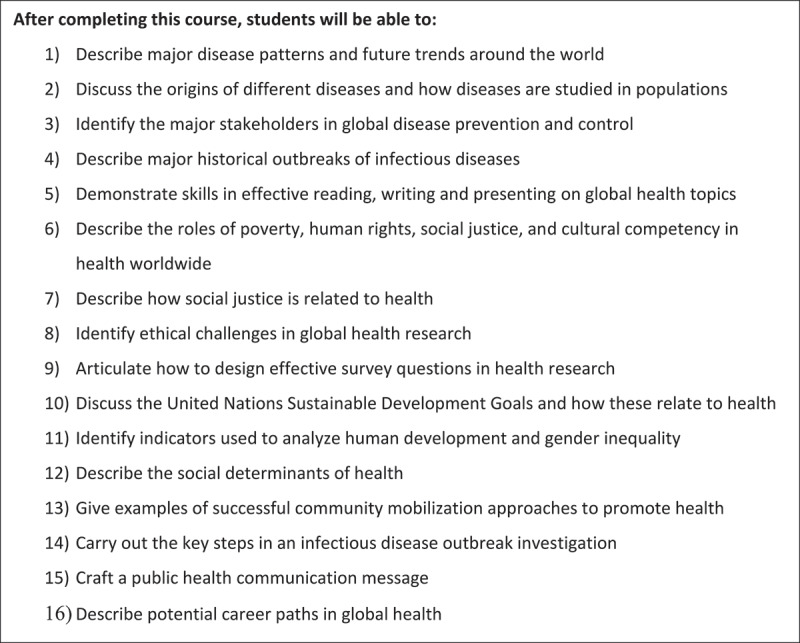
Learning objectives.

### Learning theories and approaches

In designing the course we employed both constructivism and connectivism learning theories [10,11]. First, we aim to guide students in creating their own understanding of the world around them and encourage them to incorporate their own experiences into their learning throughout the course. We developed numerous problem-based exercises using real-world examples to promote creativity and critical thinking [12]. Providing opportunities for students, especially of this age, to personally engage with the topics of the course helps them to make long-lasting, real-world connections with the material, as opposed to focusing on content. Research shows that making meaningful connections between the new knowledge and previous experiences helps to facilitate the learning process and improve student performance, including in the field of epidemiology [13]. Through constructivism we sought to recognize and promote diversity and develop active, collaborative, and applied learning [11,14]. In terms of connectivism, we sought to help students make connections to things that excite them, helping motivate them to learn and seek out more knowledge on their own. Connectivism also led us to think creatively about using gamification and simulations throughout the curriculum [15]. It encourages learning through appreciating the diversity of opinions, and capitalizing on technological resources that are particularly relevant in this digital era.

#### Activity-based and applied learning

In an effort to engage our younger learners and connect them to the world of global health, much of the course curriculum is interactive and gamified. For example, to teach disease tracking and tracing we adapted a mock mumps outbreak exercise from the Centers for Disease Control and Prevention (CDC) [16]. Working in teams, students are tasked with investigating the outbreak by interviewing several dozen ‘patients’ at USC (other staff and students in the program act as these patients with prepared scripts), calculating attack rates, and mapping locations of potential exposure. At the end of the exercise, the students host a mock press conference and invite students from other courses in the Summer @ USC program to come learn about the outbreak and what measures should be taken to control it and prevent further outbreaks. This single exercise provides training in not only basic disease surveillance, but risk communication and public health policy (e.g. recommendations for mandatory vaccination) and allows them to visualize themselves in the role of a community epidemiologist.

In relation to health behavior, students brainstorm various risky behaviors that are prevalent on campus during the Summer @ USC program. After designing their own data collection protocols, they use these protocols to collect data over one of the program weekends and return to class the following week with their own data to analyze. Past examples of such studies include surveying Summer @ USC students on use of sunscreen during weekend trips to Disneyland or the beach, assessing the impact of calorie disclosures on cafeteria menus on student selections and consumption, measuring sleep deprivation among program participants, and assessment of physical activity patterns before and during the Summer @ USC program. After analyzing their results, students prepare research posters to share with their peers.

Students also participate in numerous in-class activities and simulations, some of which also expand to other courses. For example, we have partnered with the Summer @ USC course in international relations to run simulations of a UN negotiation around vaccine development and sharing. We also meet with the Future Physician course to discuss social determinants of health and carry out comprehensive and culturally competent patient interviews. Other in-class games include Outbreak Jeopardy, Social Justice Bingo, the CDC Zombie Outbreak Investigation [17], Organizational Speed Dating (students study and quickly present their global health organization to other students), a graphic health warning design contest, and an infographic design competition. Small prizes (USC swag or Starbucks gift cards) are awarded to game winners further increasing student engagement.

### Exposure to real world careers

The course was designed specifically to provide young people with insight into the broad range of careers that are available within the global health field, with the goal of inspiring them to consider such careers in the future. To facilitate this, numerous guest speakers are invited to join the class either in person or virtually to share their professional experiences and the paths they took to arrive at their current position. Examples of such speakers include epidemiologists from the Los Angeles Department of Public Health, legal officers from the World Health Organization, physicians from LA County Hospital and Children’s Hospital of Los Angeles, research staff from Operation Smile and Mending Kids International, as well as other artists, entrepreneurs, and environmental activists. Field trips are also organized to organizations providing relevant services. For example, students learn about the prevalence of sexually-transmitted infections in Los Angeles County from County Contact Tracers and then practice contact tracing through a role-playing exercise with their peers. A County Inspector discusses food-borne illness and how restaurants are inspected. Students are given the inspection sheet and learn about the points assigned to various violation types. Past site visits include the LA County Vector Control Board (including visiting their sentinel chicken coop to test for West Nile Virus), homeless service providers, the Museum of Tolerance, environmental justice agencies, and food desert tours. Student repeatedly say that hearing from these experts in the field is one of the highlights of the course because it allows them to see the real-world application of the concepts that are presented in course modules.

### Going virtual

The most significant disruption to the course design over the past decade came in 2020 when COVID-19 forced the shift to the virtual environment. Traditionally, the course is held from 9 am-3 pm daily for four weeks in order to meet the required hours of instruction for a four-unit course. Instead, in 2020 the course was held virtually over zoom for two synchronous hours a day Monday – Friday, with up to four more hours of asynchronous materials posted daily to a course website (Blackboard). The Live Sessions often included guest speakers and provided time for student discussion and presentations. The asynchronous materials included videos, pre-recorded lectures, and learning exercises. Our core interactive group exercises, including the mock disease outbreak and the behavioral research project, were transitioned to the online format. Students continued to work together in groups, often at their own convivence based on time zones. This online format allowed for students to build community through shared discussion boards and group discussions outside of class. Through this experience, students gained experience in what it is like to work in global teams, including learning about cross-cultural collaboration and communication challenges.

## Methods for course evaluation

The course has been evaluated through two different data collection instruments. First, through an annual survey distributed to all enrolled students following the conclusion of the course, and second, through an online follow-up survey distributed to former students in Fall 2020.

### Annual course evaluation

Since 2014, USC has used the fully automated Blue course evaluation software to manage their course evaluation process, including for Summer @ USC program courses (17). All students registered in the global health course receive a link to an anonymous online survey during the final week of the class that assesses the course design, instructional practices, inclusion practices, assessment practices, and course impact. Students are asked to rank the course across a range of variables on a 4-point scale (Table 1) and can provide additional comments. A comprehensive report is provided to the course instructors following the course’s conclusion. At least 30% of enrolled students must complete the evaluation to generate a report.

**Table 1.** Course student evaluation criteria

**Course Design**	- Objectives are well explained - Assignments were related to the course objectives - I understood what was expected of me in this course
**INSTRUCTIONAL PRACTICES**	- instructors carefully explained difficult concepts, methods, and subject matter. - instructors encouraged me to do my best work - instructor encouraged questioning and discussion of course topics from the students
**INCLUSION PRACTICES**	- course materials included diverse perspectives OR applications to diverse populations - instructors used a variety of teaching approaches to meet the needs of all students - Instructors were receptive to the expression of diverse student viewpoints - instructor demonstrated sensitivity to students’ needs and diverse life experiences
**ASSESSMENT PRACTICES**	- assessments/assignments reflected what was covered in the course. - grades I have received thus far reflect the QUALITY of my performance in the course - criteria for good performance on the assignments or assessments were clearly communicated - instructor’s evaluation of my performances was constructive
**COURSE DESIGN**	I learned perspectives, principles, or practices from this course that I expect to apply to new situations This course challenged me to think critically and communicate clearly about the subject. This course provided me with information that may be directly applicable to my career or academic goals.

### Follow up evaluation

In addition to the annual survey, the course instructors designed an online Qualtrics survey to measure the impact of the course on students over time. The survey, distributed in Fall 2020, focused on collecting qualitative data on students’ motivation to learn more about global health after the course completion, subsequent academic paths taken, and eventual career choices. The survey was distributed to several dozen former students from throughout the past decade based on snowball sampling starting from those the authors had current contact information for.

## Course evaluation results

### Immediate course feedback

**Figure 1.**
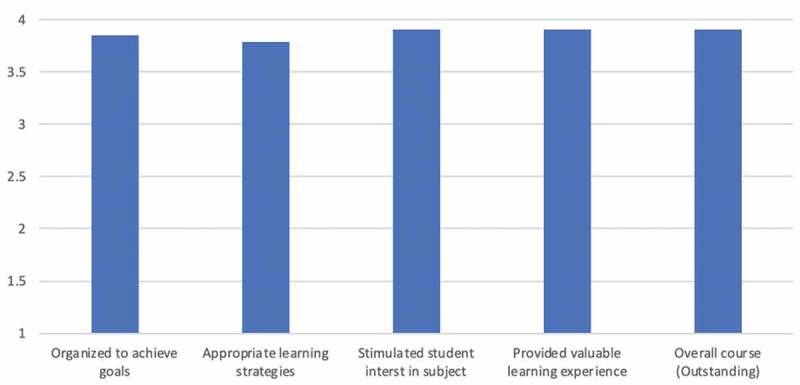
Median course evaluation scores, 2014–2021.

Blue course evaluations were available for the years 2014–2020, although the threshold for students completing the evaluation was not met in 215–2017. On average, just under 50% of students participated in the annual evaluation reports received (N = 25). On average, the course has consistently scored very high in terms of student satisfaction (Figure 1). Overall, over the past 7 years the course score ranged from a low of 3.67 out of 4 in 2019, to a perfect 4 out of 4 in 2014 and 2018. The class also scored extremely well in terms of providing a valuable learning experience and for stimulating student interest in the subject (both with an average score of 3.91 out of 4). On average, the students rated the course the lowest in terms of its overall organization to achieve clear goals (3.84) and use of appropriate learning strategies (3.79).

Statements included in the comment sections of the evaluations reflect the impact of the course in introducing students to global health and stimulating interest in the breadth of topics covered in the course. For example, one comment from 2019 reads:

Prior to signing up for the USC Summer Programs, I had never heard of global health, let alone known about the field. I remember coming to the first day of class, unsure of how to answer the question, “What is global health?” and how to draw a visual with my classmates on the definition. However, we touched on so many topics throughout the month-long course that I was able to find areas that interested me the most. – Student, Class of 2019

Another comment from 2020 reads:

One valuable aspect of this course is the diverse curriculum covered in a short period of time. We covered a lot of fascinating topics that I knew little about prior to this course.

Students also repeatedly identified guest speakers as the most valuable element of the course, emphasizing how they demonstrated numerous ways to get involved in global health in the future. Feedback on guest speakers included:

We got to hear from countless people in the global health field and I think that was the most impactful part of the program. We could see where exactly we might end up if we pursued that field. – Student, Class of 2018

The exposure to the vast field of public and global health was the most valuable thing of the course. Additionally, meeting individuals from the field through guest speakers and engaging in field trips/hands-on activities was greatly motivating and informative. Student, Class of 2019

[guest speakers] had a lot of interesting projects and jobs that were fun to hear about. These speakers demonstrated how many different ways I can get involved in global health in the future. – Student, Class of 2020

Not all students were comfortable with the interactive and game-based teaching strategies we sought to employ. For example, a student in 2012 wrote:

It was difficult to learn from the methods used by the instructors, worksheets would have communicated the lessons easier.

Notably, the switch to the online format did not result in a drop in student satisfaction with the course. In fact, in 2020, the course received a perfect score (4/4) on all measures of course impact (‘I learned perspectives, principles, or practices from this course that I expect to apply to new situations’; ‘This course challenges me to think critically and communicate clearly about the subject’; ‘This course provided me with information that may be directly applicable to my career or academic goals.’). Course design, instructional and inclusion practices also all scored above 3.8 out of 4.

### Time-lapsed feedback

Among the 22 respondents to the online Qualtrics survey, all but one student indicated that the course left them more interested in global health than when they began the class (the one exception was already very interested and remained so). Years after completing the course, students pointed to the background they acquired in epidemiology, social determinants of health, and global health governance as especially valuable in hindsight. They also continued to highlight the impact of guest speakers at the time and in the following years. Over one third (n = 8) of the former students responded that they had ‘decided to pursue a global health or related field as a result of the course’, while a handful more indicated that while they did not decide to go into the field, the course had influenced their career paths. Numerous students reflected on the benefit of the course in terms of the way they view the world. Example feedback from the students in regard to the lasting impact of the course on their career included:

It really introduced me to the field of healthcare and inspired me to pursue it as a career. It really shaped my perceptions of others - to look at, to understand, and to approach different cultures in a way to fully encompass the adequate delivery of healthcare such that patients and communities can learn and adapt their own ideals and social norms. – Student, Class of 2011

When I took the course, I was just starting my senior year of high school and unsure of what health related field I was truly passionate about but this course and subsequent mentorship from the faculty sparked an interest. I was inspired to pursue a college degree in the public health and MPH in epidemiology, which I am currently in the midst of completing at USC! – Student, Class of 2014

This course made me further investigate and pursue public/global health in high school, and after more experiences I ultimately decided to pursue this field in college. In addition, this course made me rethink my then-desire to go to medical school and while I am still unsure whether I want to pursue an MD I have put much more thought into this decision than I likely would have otherwise. – Student, USC Class of 2018

Notably, even students who indicated that they did not go on to pursue global health or a related field indicated that the course has still impacted their life, and in many cases, continues to do so. Examples of this type of feedback included:

I’m not directly related to the field, but a lot of what I deal with in urban planning are public health issues. – Student, USC Class of 2011

Since the course, I have continued and deepened volunteer work in the medical sector in Peru with my family. - Student, USC Class of 2010

Although I did not pursue further studies in global health, [in the class] I learned to collaborate better with others, especially since there was a wide array of backgrounds in the class (backgrounds including socioeconomically and ethnically). – Student, Class of 2010

The course introduced me to the field of public/global health and made me aware of how our health is shaped by social determinants. – Student, Class of 2014

## Discussion and recommendations

The results of the course’s immediate and time-lapsed evaluations indicate that the course is generally well-designed and implemented to introduce young learners in global health and interest them in the field. While also strong, the student evaluations do leave some room for improvement regarding the courses’ instructional approaches and student assessment strategies. Lower evaluation scores in terms of instructional practices may in part reflect students’ past experiences in high school. For example, students who preferred more traditional approaches such as worksheets were likely not used to anything else. Students reflected some level of anxiety towards our courses’ innovative design and lack of clear assessment rubrics assigned to exercises and assignments. Discomfort with our evaluation practices also likely reflected high school student’s expectation for very specific grading rubrics that, when followed precisely, result in perfect grades – a perceived necessity for high performing high school students seeking admittance to the most competitive US colleges and universities and those most often enrolled in the Summer Program. It can be an adjustment for students to accept the harder and often more subjective nature of grading at the collegiate level based on the quality of the content and argumentation and to accept anything less than an A grade. To help relieve some anxiety, we have limited the number of assignments that contribute to the final grade, making most assignments simply completion based to encourage the students to take risks without concern for whether it is perfect and have provided example assignments to help guide the students in terms of our expectations. We have had to balance making our assessment practices more acceptable to the students against the university requirements to demand college-level reading assignments, homework, exams, and formal student assessment for the students to receive college course credit.

Another challenge we have faced is determining the right balance between breadth and depth in the course materials. While it is important to solidify key concepts, students clearly appreciated learning about diverse topics relating to global health. We recommend designing content using a student-centered approach that includes scaffolding with short lectures combined with engaging active learning exercises that are appropriate for learning in this age group, as has been highlighted by others [12,13]. Notably, we did not receive any specific instruction in how to teach 14–17-year-old learners. The presumption is that we will adapt our existing college level course to the summer program format. While we recognized that we needed to make adaptions to keep our young learners engaged, we likely would have benefited from some support in this effort from educators with such experience and specific instruction on how to best design and communicate our assessment strategies the students.

We sought from the start to design the course materials to reflect the interdisciplinarity of the field of global health. We also found it crucial to bring diverse voices and viewpoints to support the course’s focus on social justice issues, and to promote diversity and inclusion. While in class exercises focused on these areas, the field trips to sites such as a homeless shelter and environmental racism organizations were essential in helping students gain an appreciation for the complexity of these social problems. These experiences also helped to humanize these issues, putting a face to homelessness or migration. Many students had emotional responses to some of the field trips, guest speakers, and even course materials such as film clips. These were opportunities for students to develop more empathy and tolerance. We found that being able to debrief and encourage open dialogue about students’ responses helped them to connect on a deeper level with the materials and with one another. Building in time for reflection should be considered an integral part of learning in any global health course. Student reflexivity and critical thinking can help better solidify the lessons learned and their application beyond the classroom [13].

**Image 1.**
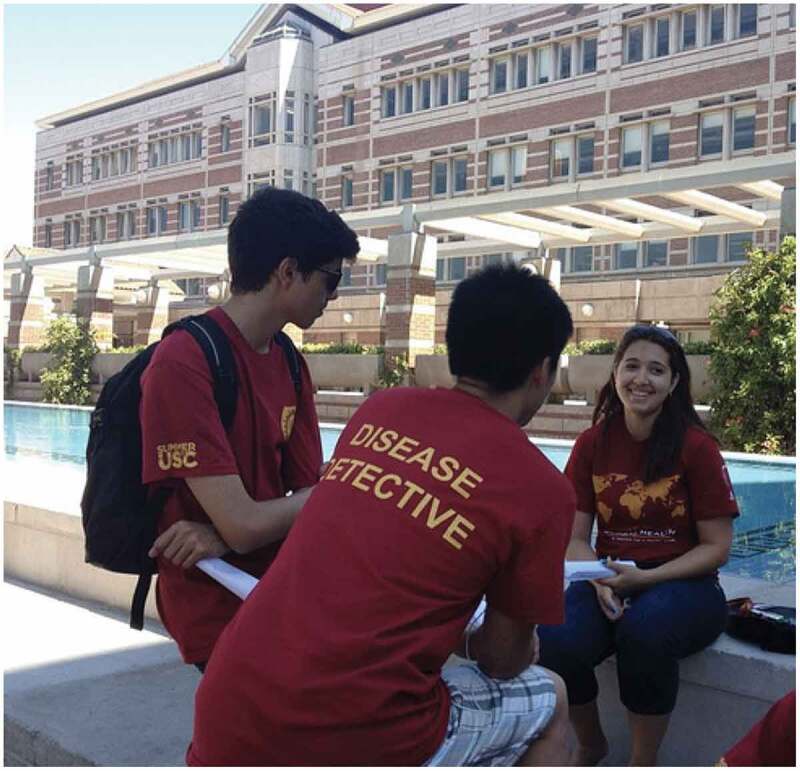
Summer high school students carry out a mock disease outbreak investigation on the USC campus wearing their course t-shirt.

As mentioned, keeping the curriculum engaging and relevant for this age required extra thought and preparation. We were fortunate to have an instructional supply budget provided by the Summer @ USC program. This budget allowed us to purchase supplies such as posterboard and markers, small prizes for games, books, and food for special events, like the graduation party. These funds were also used for field trips, as well as to give small donations to the guest speakers or to the community-based organizations that hosted field trip visits. We also provided t-shirts and university ‘swag’ that helped to build community among students (Image 1). Other universities running similar programs to engage young high school learners in global health should also consider providing instructional materials/lab funds for instructors to use in delivering their courses. Such fees could be included in the tuition of paying students. Of course, these engagement strategies were not relevant to when we moved to the online format, although the need to actively engage learners (and keep them off of other screens) was more critical than ever. We incorporated both formative and summative assessment to build critical thinking skills while using technology to enhance the user experience [18–20].

Another key lesson we have learned is the importance of building community among students very quickly. The course flows much better when we have built group exercises into the materials at the beginning with the purpose of helping students to break the ice and form connections. This community feeling leads to richer discussions and allows young students who may be intimidated to voice their opinions and experiences in the new setting. Notably, the richness of the discussions also depends on the diversity of the experiences within the community. One observation we have made as instructors is the privileged nature of the student body participating in the course. As mentioned, programs such as Summer @ USC are expensive and universities should find ways of providing these early global health training opportunities to more diverse and underprivileged youth who are needed to diversify the global health (and other sectors) workforce. At USC, for several years we were able to welcome a couple low-income students to join the course through a separate year-long global health lab experience for talented local high school students [21]. More recently, USC’s Provost’s Pre-College Military Scholarship has provided highly motivated high school students from military families full scholarships to join the program [22]. The inclusion of these students broadens representation among the students and helps to enrich classroom discussions, especially about health disparities. The inclusion of international students has also been valuable; international students bring different cultural and contextual perspectives that help to liven course discussions and broaden students’ worldviews.

Finally, the course’s promotional materials must be carefully considered. Because most students at this age are unfamiliar with the field of global health, promotional material should be written in a way that is concise and descriptive but also attractive to students. In the first couple of years of the program, we struggled to recruit large numbers of students and several students mentioned that the course description was too academic and made it sound boring and too challenging compared to other courses. It is also important to make the course relevant and relatable to students exploring many fields, as opposed to only those interested in health-related careers. And of course, to appeal to students, the description must also highlight the fun, interactive aspects of the course. Box #2 shows the final course description for the 2020 course.

**Box #2.**
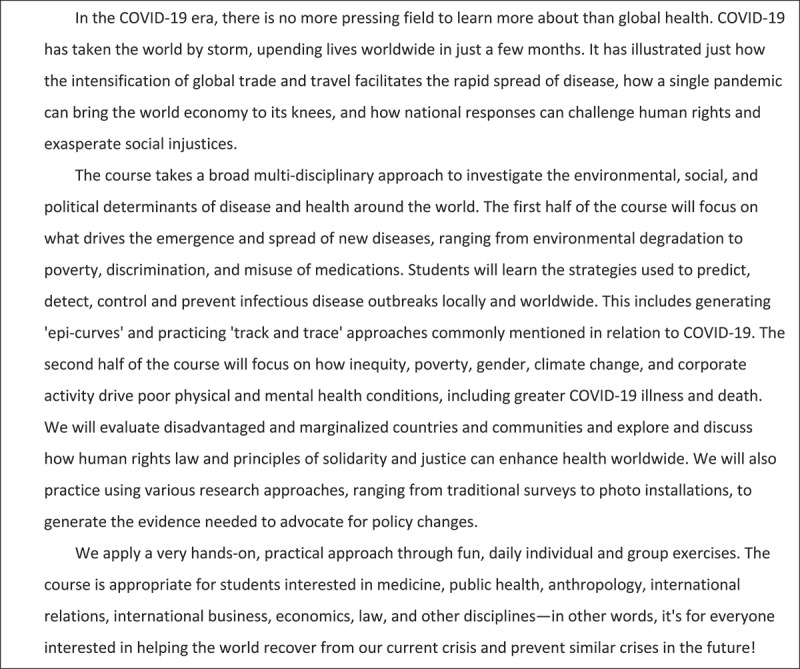
Description of 2020 course.

Our course evaluation faces several limitations. We continue to receive a low response rate from participating students. After recognizing that students were failing to complete the assessment, dedicated time was allocated in class during the final session for students to log in and fill it out. Still, over half of students refrain from completing the survey. To address this, we may need to incorporate proof that students have completed it into their formal participation grade. Getting more reluctant evaluators to complete the survey may result in lower evaluations, although experience shows that unhappy students do tend to be motivated to provide feedback. It is more likely that unresponsive students are content and have very little feedback, so they chose to skip the assessment. Our evaluation is also not course specific, so we don’t get feedback on unique lessons, although as seen from the evaluation comments specific elements of the course are repeatedly mentioned by students. Tracking young students over time is also difficult and led to clustering of students within specific years that have remained in touch with each other. This may indicate they were part of classes that were particularly well bonded and had stronger relationships with the instructors, thus resulting in more positive feedback on the course and its long-term impact. Regardless, the evaluation data we have been able to collect does indicate that the course is generally well received and has had a lasting impact on some former students.

## Conclusions

An increase in global health training opportunities for high school students is warranted. Given strong student interest, more high school programs should consider offering global health curriculum and universities should extend their undergraduate programs to include programs that reach potential future students with global health content, such as through their summer programs. These programs can serve as a pipeline to majoring or minoring in global health once students reach the university level [5,13,18].

The COVID-19 pandemic has illustrated to the entire world the need to increase our collective capacity to respond to pressing global health challenges. This will require the best and brightest young minds to dedicate their careers to addressing health inequities, correcting social injustice, and solving biological quandaries. Introducing students to the field of global health early in their academic careers can result in greater motivation to pursue these pressing topics and ensure that even if they don’t, they have the background necessary to understand the relationships between health and poverty, governance, and justice. As shown in the example of USC’s high school summer course in global health, engaging and interactive curriculum can be designed that entertains young learners, while teaching them fundamental skills and leaving a lasting legacy on their future career decisions.

## Paper context

Global health education, which goes beyond core public health competencies to include social justice, is crucial to ensuring the next generation of global health professionals are poised to tackle the pressing issues of our time. This paper reviews the development and implementation of a global health course targeting young secondary school students and reports the impact of the course on student educational and career outcomes up to ten years after completing the course.
